# A young woman with renal colic presenting with urogenital anomaly: a case report

**DOI:** 10.4076/1757-1626-2-8225

**Published:** 2009-08-24

**Authors:** Konstantinos Michalakis, Dimitrios-Anestis Moutzouris

**Affiliations:** 1 Endocrine Department, National Institutes Of HealthBethesda, Maryland, 20815USA; 2 2^nd^ Department of Internal Medicine, “Asclepieion” General HospitalAthens, 16673Greece

## Abstract

A 26-year-old female presented with a two-week history of right flank pain. She underwent abdomen ultrasound which revealed moderate pelvicalyceal dilatation in the right kidney and proximal ureter with no apparent cause. Intravenous pyelography showed a fish-hook (reversed ‘J’) shape of ureter. No renal tract calcification was noticed. The findings were consistent with that of a retrocaval ureter.

## Case presentation

A 26-year-old Caucasian female presented in the emergency room complaining of a two-week history of right flank pain. Her past medical history was unremarkable but included an episode of pyelonephritis two years ago. Physical examination was unremarkable: normal pulmonary examination, heart murmurs S1, S2 +0 bruits, neurologic examination normal - negative focal signs, GI examination normal, tender non sensitive abdomen, normal bowel sounds, except for tenderness at the right costophrenic angle. Routine laboratory tests were within normal limits. Blood panel was normal, biochemical panel showed normal electrolytes and renal function, liver enzymes and amylase were within the normals. Urinalysis showed no significant findings and the urine culture was sterile.

The patient underwent abdomen ultrasound which revealed moderate pelvicalyceal dilatation in the right kidney and proximal ureter with no apparent cause. The kidney-ureter-bladder X-ray did not reveal abnormal findings (Panel A). However, intravenous pyelography showed a right hydronephrosis which had a characteristic fish- hook (reversed “J”) shape of ureter (Panel B, arrow). In addition, there was medial deviation of the ureter. No renal tract calcification was noticed. The findings were consistent with that of a retrocaval ureter [1,2], which was confirmed by computed tomography (CT) [3].

The patient is currently followed in renal clinic and a surgical correction would be performed in the near future.

## Conclusion

We believe that the submitted manuscript and related images have educational value for medical students, residents, and practicing clinicians. The physician should bear in mind the variable causes, clinical pictures and laboratory findings of a renal colic, as well as anatomical variations of the urogenital system, when evaluating a patient with such a clinical manifestation, shortening the time to diagnosis and improving the outcome.

**Figure 1.**
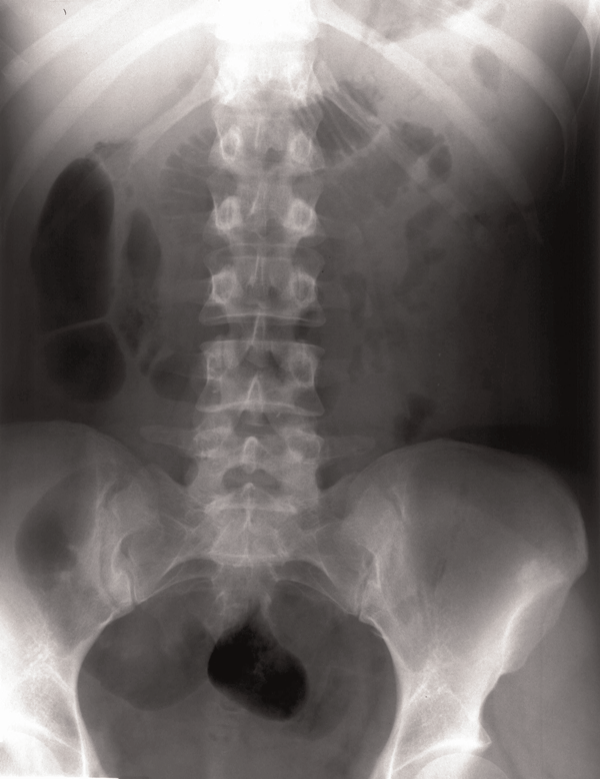
The kidney-ureter-bladder X-ray: no abnormal findings.

**Figure 2.**
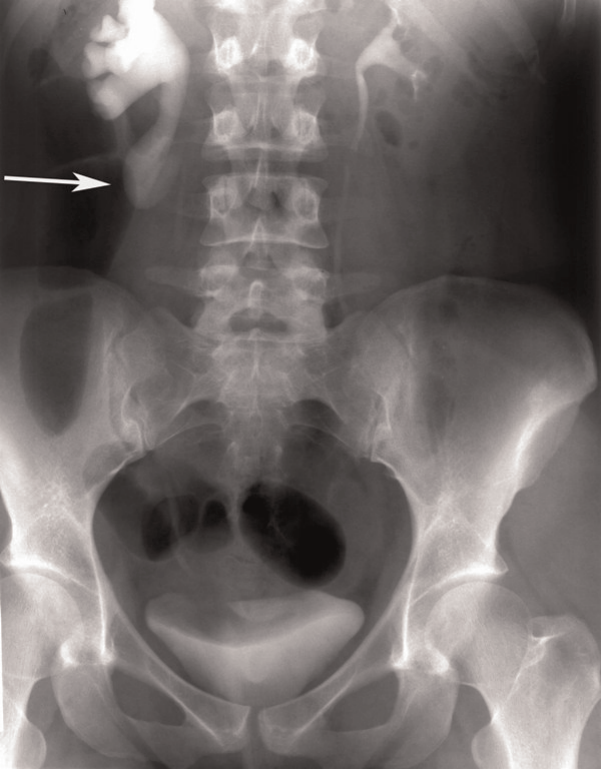
Intravenous pyelography showed a right hydronephrosis which had a characteristic fish-hook (reversed “J”) shape of ureter (arrow).

## Abbreviations

CT: computed tomography
GI: gastrointestinal tract
